# The effect of olsalazine of chinese generic drugs on ulcerative colitis induced by dextran sulfate sodium salt in BALB/c mice

**DOI:** 10.1590/acb382923

**Published:** 2023-08-21

**Authors:** Zheng-Yong Yu, Yu-Sheng Xu, Miao Tang, Wen-Feng Xin

**Affiliations:** 1 Wenshan University – College of Notoginseng Medicine and Pharmacy – Yunnan – China.; 2 Hunan Agricultural University – Agricultural School – Hunan – China.; 3 Dali University – Yunnan Provincial Key Laboratory of Entomological Biopharmaceutical R&D – Yunnan – China.

**Keywords:** Drugs, Generic, Colitis, Ulcerative, Dextran Sulfate

## Abstract

**Purpose:**

To explore effect and mechanism of olsalazine of Chinese generic drugs on ulcerative colitis induced by dextran sulfate sodium salt (DSS) in BALB/c mice.

**Methods:**

The mouse model of ulcerative colitis was induced by free drinking of 3% (w/v) DSS aqueous solution for seven days. The mice were treated with olsalazine (0.6 g·kg^-1^) of Chinese generic drugs. The therapeutic effect of olsalazine on ulcerative colitis mice was evaluated by measuring disease activity index (DAI), colonic mucosal injury index (CMDI), histopathological score (HS), and detected the expression levels of interleukin (IL)-2, IL-10, tumor necrosis factor-α (TNF-α), interferon-γ (IFN-γ), IL-1β in serum and IL-7, IL-17, IL-22, epidermal growth factor (EGF), transforming growth factor β1 (TGF-β1) in colonic homogenate of mice.

**Results:**

Olsalazine significantly increased the contents of IL-2, IL-10, IL-22, TGF and EGF in ulcerative colitis rats, and significantly decreased the scores of DAI, CMDI, HS and the contents in IL-7, IL-17, TNF-α, IL-1β and IFN-γ when compared with the model group. It improved the degree of colonic lesion in ulcerative colitis mice.

**Conclusions:**

It was suggested that olsalazine has a therapeutic effect on ulcerative colitis induced by DSS in mice, and the mechanism may be related to the increase of IL-2, IL-10, IL-22, TGF, and EGF and the decrease of the expression of IL-7, IL-17, TNF-α, IL-1β, and IFN-γ.

## Introduction

Both ulcerative colitis (UC) and Crohn disease (CD) belong to the category of inflammatory bowel disease. UC is a common chronic non-specific intestinal disease, which mainly involves colon, rectal mucosa, and submucosa, showing phased and diffuse distribution^1^. The clinical manifestations are abdominal pain, diarrhea, and mucous bloody stool, and UC may be accompanied by serious complications. UC has the characteristics: high acute fulminant mortality, high rate of chronic persistent cancelation, and easy recurrent attacks. It has been defined as a precancerous disease and listed by the World Health Organization as a refractory disease in the world^2^.

With the great changes in people’s lifestyle concerning eating habits and environment, the disease spectrum has also changed. The increasing incidence of UC year by year is the manifestation of this change. Based on the case statistics of many hospitals in China, it is inferred that the incidence rate of UC in China is 11.6/100,000, while in Western countries the incidence rate is 10/100,000–20/100,000, and the prevalence rate is 100/100,000–200/100,000. The age of onset of UC is between 15 and 40 years old, and there may be a second peak between 50 and 80 years old. There is no significant difference in incidence between men and women, and there is family aggregation^3^–^5^. The etiology of UC is complicated, and its pathogenesis is still unknown. It is generally believed that its pathogenesis is closely related to genetic gene, environment, infection, immunity, and so on^6^–^9^.

The animal models used for anti-UC drug screening mainly include chemical stimulation animal model, genetic engineering animal model, tissue or cell transplantation animal model, spontaneous animal model, and traditional Chinese medicine (TCM) syndrome animal model at present. The dextran sulfate sodium salt (DSS) of chemical reagent stimulation animal model is widely used in experimental research because of its advantages, such as easy to make, low cost, strong maneuverability, good repeatability, among others.

DSS was used to induce acute UC in BALB/c mice in this study, and the treatment of UC mice was by intragastric administration of olsalazine of Chinese generic drug. It was observed the effect of olsalazine on UC mice and to explore the mechanism of olsalazine on DSS-induced UC in BALB/c mice from the changes of general condition, body weight, disease activity index (DAI) score, organ index, colon colonic mucosal injury index (CMDI) score, serum antibody, colon pathological, and biochemical indexes.

## Methods

### Reagents and equipment

DSS (Shenzhen Lijing Co., Ltd., China), olsalazine (Tianjin Lisheng Pharmaceutical Co., Ltd., China), formaldehyde solution, disodium hydrogen phosphate, and sodium dihydrogen phosphate were procured from Tianjin Fengchuan Chemical Reagent Technology Co., Ltd., China. Sodium chloride injection (Guizhou Tiandi Pharmaceutical Co., Ltd., China), erythrocyte lysate (Thermo Fisher Co., Ltd., China), phosphate-buffered saline (PBS) (Solarbio Co., Ltd., America), fetal bovine serum (Zhejiang Tianhang Biotechnology Co., Ltd., China), occult blood kit, mouse interleukin (IL)-1β enzyme linked immunosorbent assay (ELISA) kit, mouse IL-2 ELISA kit, mouse IL-7 ELISA kit, mouse IL-10 ELISA kit, mouse IL-17 ELISA kit, mouse IL-22 ELISA kit, mouse transforming growth factor (TGF) ELISA kit, mouse epidermal growth factor (EGF) ELISA kit, mouse tumor necrosis factor-α (TNF-α) ELISA kit, and mouse interferon-γ (IFN-γ) ELISA kit were procured from Nanjing Jiancheng Biological Engineering Research Institute, in China. Electronic analytical balance (AL204-IC, Mettler-Toledo Instruments Co., Ltd., China), high-speed centrifuge (HC-3018R, Anhui Zhongke Zhongjia Scientific Instrument Co., Ltd., China), electronic balance (Forerunner CP214, Ohaus Instruments Co., Ltd., China), medical image analysis system (BI-2000, BD Co., Ltd., America), enzyme labeling instrument (SpectraMax M5, Molecular Devices, America).

### Preparation of main reagents

#### Preparation of 3%DSS solution

120-g DSS were dissolved in 4-L hot-pressed ultra-pure water to obtain 3% DSS solution (currently used).

#### Preparation of PBS buffer (pH = 7.4)

Precisely 8-g NaCl, 0.2-g KCl, 1.44-g Na_2_HPO_4_·12H_2_O, and 0.24-g KH_2_PO_4_ were put into a flask, then 1,000-mL ultra-pure water was added. The mixture was shaken well and sterilized with high pressure steam (121°C, 15 min) (storing at 4°C).

#### Preparation of 10% neutral formalin

Precisely 6-g Na_2_HPO_4_, 4-g NaH_2_PO_4_, and 50-mL formaldehyde solution was inserted into a 500-mL volumetric flask, and 500-mL ultra-pure water was added.

### Establishment and grouping of models

Seventy-two healthy male BALB/c mice were fed adaptively for one week. Twenty-four mice were randomly selected as the normal group to drink ultra-pure water, and to the rest 3% (w / w) DSS solution was given freely for seven days, and all the mice were scored by DAI. The mice with very mild inflammation were excluded, and the other mice were randomly divided into model group and olsalazine group (0.6g kg^-1^), with 24 mice in each group. At the end of the model, the treatment group was given olsalazine according to 0.1 mL·kg^-1^·d^-1^, while the model control group and normal control group were given normal saline 0.1 mL kg^-1^·d^-1^ intragastrically for 10 days.

### Observation of indicators

#### Observation of general state

The changes of body mass, drinking water, diet, and fecal characteristics of mice in each group were observed and recorded during the experiment.

#### The score of disease activity index

**Figure 1:**
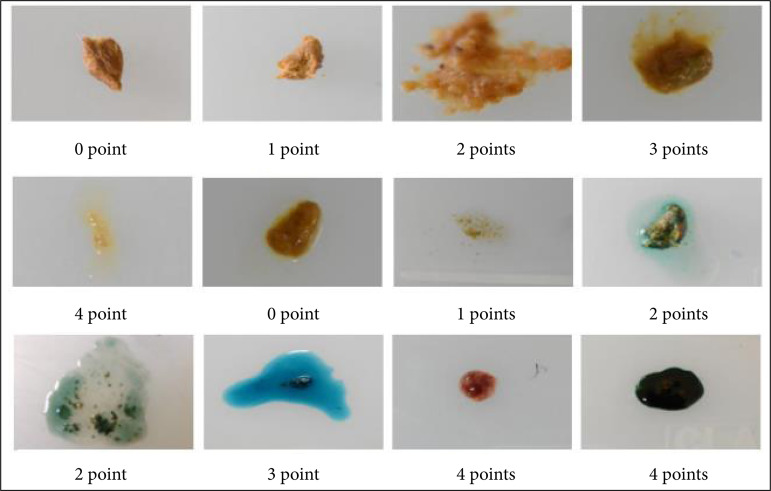
Scores of fecal traits and occult blood.

Source: The Hamamoto standard^10^.

On the first, fourth, seventh, eighth, tenth, 14^th^, and 17th day of the experiment, the normal group and the model group referred to the Hamamoto standard^10^. The mice were weighed, the fecal traits and fecal occult blood were observed, and the DAI scores of the mice were evaluated. The disease activity was evaluated by DAI on the eighth, 11th, 14th, and 17th day in the olsalazine group, and the DNA score criteria are shown in Table 1. Fecal traits and occult blood score are shown in Fig. 1.

**Table 1:** Evaluation of disease activity index (DAI).

Score of DAI	Stool consistency	Occult blood test	Weight loss* (%)
0	Normal	Negative (-)	< 1
1	Normal-sparse stool	Weak positive (+)	1–5
2	Sparse stool	Positive (++)	5–10
3	Sparse stool diarrhea	Strong positive (+++)	10–15
4	Diarrhea	Bloody stool	≥15

Normal feces: formable faeces; sparse stools: sparse faeces that do not stick to the anus; diarrhea: liquefied feces that stick to the anus;

* weight loss ratio range, excluding the first number, including the latter number. Weight loss (%): (body weight at a certain point after modeling-premodeling weight)/premodeling weight) × 100%; DAI scores: weight loss scores + stool trait scores + occult blood scores.

#### Colonmucosa damage index

The removed colon was laid flat on white paper, and the colonic mucosal injury was observed and scored according to standards of Ekström et al.^11^ and Luk et al.^12^ in Table 2.

**Table 2:** Colonic mucosal injury index scoring criteria.

Score	The mucous membrane of the colon
0	No damage
1	Mild hyperemia and edema, smooth surface, no erosion, or ulcer
2	Hyperemia and edema, rough mucous membrane, granular sensation, erosion, or intestinal adhesion
3	Severe hyperemia and edema, necrosis, and ulcer formation on the surface, thickening of intestinal wall or necrosis and inflammatory polyps on the surface
4	Severe hyperemia and edema, mucosal necrosis and ulcer formation, whole intestinal wall necrosis, death caused by toxic megacolon

Source: Ekström et al.^11^ and Luk et al.^12^.

#### Organ index in mice

The liver, spleen, lung, thymus, and colon were taken, and the organ index was calculated (Eq. 1).


Organ index=Organ mass(mg)/Mouse body weight(g)

#### Observation on the pathomorphology of colon

The longitudinally sectioned colon was cut into two halves, wrapped, fixed, and preserved in 10% neutral formalin solution. The pathological sections were observed under light microscope after hematoxylin and eosin (HE) stained, and the colon histopathological score (HS) was performed according to Ekström^11^ standards (Table 3). The HS re was diagnosed by Yan Changbao, an attending physician in the Department of Pathology of the people’s Hospital of Dali Bai Autonomous Prefecture in China.

**Table 3:** Scoring criteria of histopathology*.

Score	Epithelial cell	Degree of inflammatory cell infiltration
0	Normal form	No infiltration
1	Loss of a small number of goblet cells	Infiltrate into the basal layer of the crypt
2	Massive loss of goblet cells	Infiltrate into the muscular layer of the mucosa
3	Loss of a small number of crypt cells	The infiltration penetrated into the muscular layer of the mucosa, accompanied by mucosal thickening and edema
4	Massive loss of crypt cells	Infiltrate into the submucosa

* Colonic histopathological score: “epithelial cell” score + “inflammatory cell infiltration” score. Source: Ekström et al.^11^.

#### Detection of related biochemical factors in serum and colonic mucosa

The blood extracted from eyeballs of mice was placed at 4°C for 4 hours, and the supernatant was obtained by centrifugation at 3,000 rpm (4°C) for 10 min. The other half of the longitudinally dissected colon was made into 10% homogenate in an ice bath, and the supernatant was obtained by centrifugation at 3,000 rpm (4°C) for 10 min. According to the instructions, the contents of related biochemical factors in serum and colonic mucosa were determined by ELISA method, and the data were calculated by ELISACalc.

#### Determination of lymphocyte subsets

One-hundred-μL spleen cell suspension (10^6^ spleen cells) was added to the flow tube, and the corresponding mixed fluorescent antibody was added for FACS Calibur detection. The ratio of T lymphocytes (CD3^+^) to single spleen cells and the proportion of Th cells (CD3^+^CD4^+^CD8^-^), Tc cells (CD3^+^CD4^-^CD8^+^) and Treg cells (CD3^+^CD4^+^CD25^+^) to T lymphocytes in spleen were analyzed.

### Statistical analysis

Statistical Package for the Social Sciences (SPSS) 23.0 and GraphPad Prism 5.0 were used to describe and analyze the data. Rank sum tested was used to compare the rank data, and the concentrated trend and discrete trend are expressed by median (M) and quartile (QR) spacing, respectively. The measurement data are expressed as *X* ± *S*, the data onto normal distribution and uniform variance are analyzed by t-test and one-way analysis of variance (ANOVA), and the data that are not consistent with normal distribution are tested by rank sum test. The continuous data were analyzed by repeated analysis of variance and univariate analysis of variance. Least-significant difference (LSD) tested was used for pairwise comparison between groups. *P* < 0.05 was used as the standard of statistically significant difference.

## Results

### Changes in general physical signs

In the first three days of the experiment, the body weight of the model mice changed little, and on the third day, mice began to develop bloody stools, loose stools, severe weight loss and reduced water consumption. On the seventh day, mice showed mental malaise, hematochezia or diarrhea, curled up in piles and dull color. The intestinal wall of the mice was swollen and congested, but no obvious ulcer was found in the naked eye. On the tenth day, the body weight of the mice slowly recovered, the drinking water returned to normal, the symptoms of diarrhea were mild, the hematochezia decreased or even disappeared, but the activity did not increase. The activity and body weight on the 14th day increased, and the coat color returned to a lighter luster. At the end of the model, after administration of oxalazine (0.6 g·kg^-1^), the weight loss of mice slowed down, followed by weight gain, coat color gradually returned to luster, and the amount of exercise increased.

### Impact of disease activity index score

**Figure 2:**
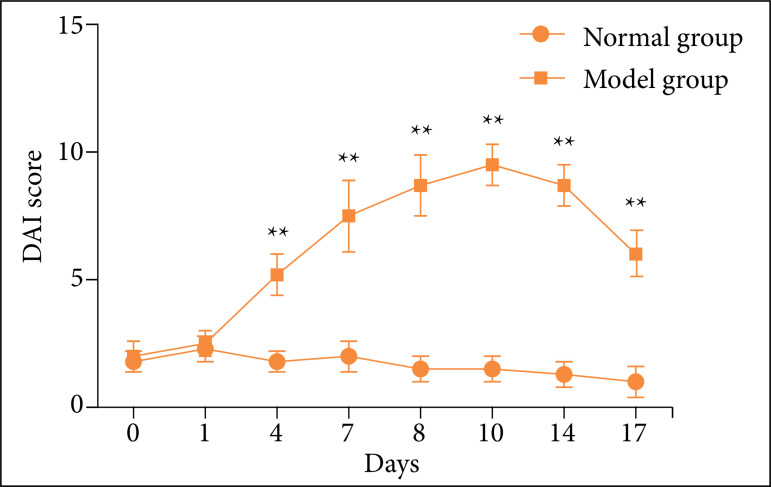
Changes of disease activity index (DAI) score in mice at different time. Compared with the normal group, *P < 0.05, **P < 0.01. Compared with the model group, ^∆^ *P* < 0.05, ^∆∆^ *P* < 0.01.

Source: elaborated by the authors.

The feces of mice in the normal group were basically formed, and the feces of some mice were loose or weakly positive for occult blood. However, with the increase of days of drinking DSS, thin stools, bloody stools, and weight loss occurred; weight gain, stool score, occult blood score decreased after administration. The scores of DSS-induced acute UC mice increased gradually on the first, fourth, seventh, eighth, and tenth day DAI. The 10^th^-day DAI score reached the peak in the experiment. Compared with the normal group, the DAI score of each model group was significantly higher than that of the normal group at different time points (P < 0.01). The DAI score of the mice in the drug group was significantly lower than that in the model group at different time periods of treatment (P < 0.01). The results are shown in Fig. 2.

### Changes of colonic length and colonic mucosal injury index

**Figure 3:**
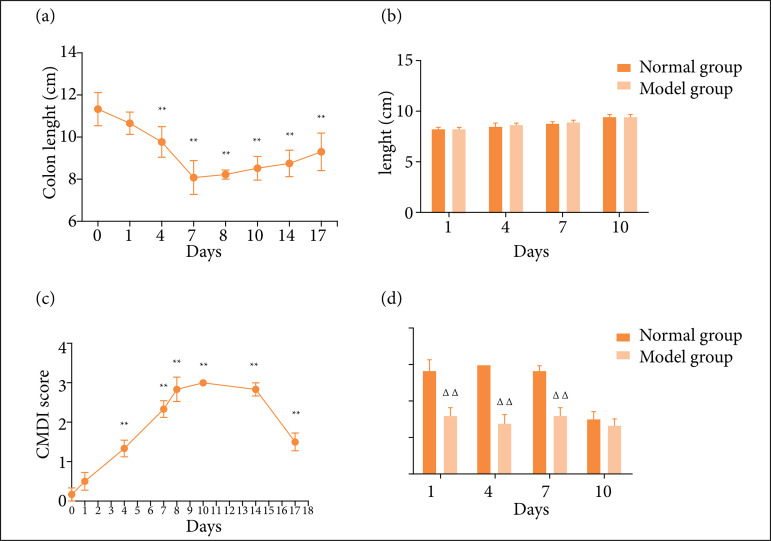
Changes of colonic length and colonic mucosal injury index (CMDI) in ulcerative colitis mice at different time points. **(a)** The colonic length of mice at different time points during the modeling period. **(b)** Colonic length of mice at different time points during administration. **(c)** Changes of CMDI score of mice at different time points during modeling. **(d)** Changes of CMDI score in mice at different time points during administration. Compared with the normal group, *P < 0.05, **P < 0.01. Compared with the model group, ^∆^ *P* < 0.05, ^∆∆^ *P* < 0.01.

Source: elaborated by the authors.

The colon length of mice in the normal group changed little, but the colon length in the model group decreased gradually with the increase of days of drinking DSS, and the length of colon recovered from the eigthth day (Fig. 3a). On 0 day, the general morphology of mouse colon was normal, the mucosa was smooth, and no mucosal hyperemia, edema, erosion, and ulcer were found. With the increase of days of drinking DSS, colonic inflammation, colonic mucosal congestion and edema, intestinal wall thickening and erosion, ulcer, oval ulcer, marginal hyperemia, and edema, CMDI score also showed an upward trend. After stopping drinking DSS, it gradually recovered and reached the peak on the tenth day CMDI (Fig. 3c). Compared with the model group, when oxalazine was given for on, four and seven days, the CMDI of mice decreased significantly (P < 0 01). At the 17^th^ day, there was no significant difference in CMDI between the model group and each treatment group (P > 0.05), and there was no significant change in colon length from the beginning to the end (Figs. 3b and 3d).

### Score of colonic histopathology in mice

**Figure 4:**
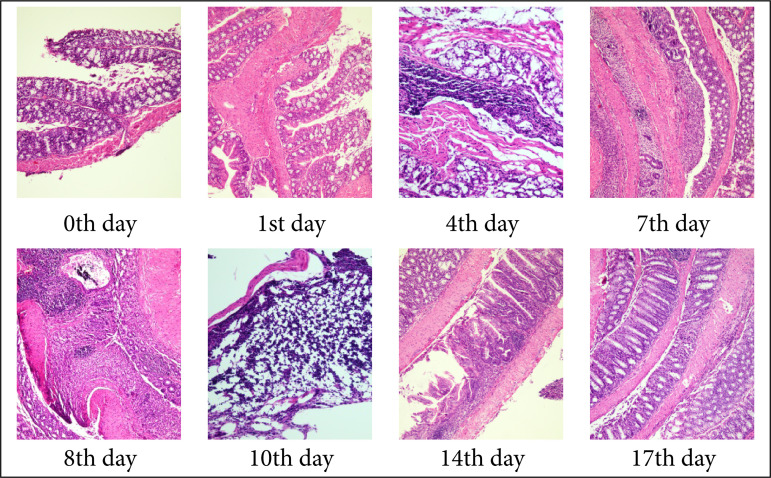
Histopathological score picture of colon of model mice at different time points after modeling (hematoxylin and eosin, 10 × 10).

Source: elaborated by the authors.

As shown in Fig. 4, a small number of goblet cells were lost, and a small number of inflammatory cells infiltrated in normal mice. In the model group, with the increase of modeling days, there was a large area loss of goblet cells, different degrees of crypt defect, and a large area of inflammatory cells infiltrated into the mucous membrane and submucosa. After discontinuation of DSS, the defects of goblet cells and crypt cells gradually recovered, and the degree of inflammatory cell infiltration decreased, but it was still significantly different from that of the normal group. The HS showed that, compared with 0 day, the colonic HS of mice at each time point increased in varying degrees, and the HS of the eighth-, tenth-, 14th-, and 17th-day model mice was significantly higher than that of 0 day (*P* < 0.01). On the 10th day HS reached the peak, and then showed a downward trend, as shown in Table 4.

**Table 4:** Changes of colonic mucosal injury index in model mice at different time points.

Number of days	Quantity	HS score									P-value (rank sum test) Compared with 0 day
		0	1	2	3	4	5	6	7	8	
0	6	0	0	6	0	0	0	0	0	0	-
1	6	0	0	3	3	0	0	0	0	0	0.180
4	6	0	0	2	2	0	1	0	0	1	0.065
7	6	0	0	1	0	0	0	5	0	0	0.015
8	6	0	0	0	0	0	0	1	5	0	0.002
10	6	0	0	0	0	0	0	1	1	4	0.002
14	6	0	0	0	0	0	0	5	0	1	0.002
17	6	0	0	0	3	1	0	2	0	0	0.002

*α = 0.05 and the α’of Benferroni correction method is 0.007. Source: elaborated by the authors.

**Figure 5:**
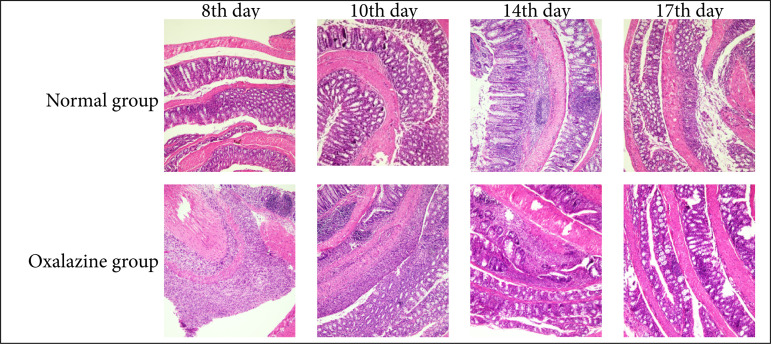
Changes of histopathological score of ulcerative colitis mice at different time points in each administration group (hematoxylin and eosin, 10 × 10).

Source: elaborated by the authors.

Compared with the model group, the mice in the treatment group showed crypt blood stool, goblet cell repair and less inflammatory cell infiltration, which showed that the scores of HS decreased with the increase of administration days. The HS decreased significantly on the 14th day, but there was no significant difference on the 17th day, as shown in Figs. 4 and 5.

### Changes of T cell subsets in model mice at different time points

**Figure 6:**
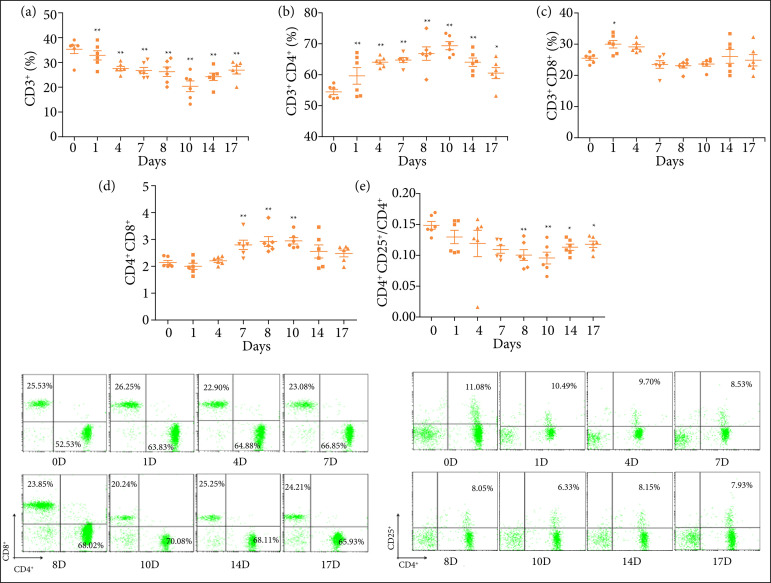
Changes of T lymphocyte subsets in splenocytes of model mice at different time points. **(a)** CD3+ occupies cellular level. **(b)** CD3+CD4+ occupies cellular level. **(c)** CD3+CD8+ occupies cellular level. **(c)** CD3+CD4+/CD3+CD8+ occupies cellular level. **(d)** CD3+CD4+CD25+/CD3+CD4+ occupies cellular level. **(f)** Flow cytometric analysis of CD4+ and CD8+ T cells in model mice at different time points. **(g)** Flow cytometric analysis of CD4+CD25+T cells in the spleen of model mice at different time points. Compared with the normal group, *P < 0.05, **P < 0.01. Compared with the model group, ^∆^ *P* <0.05, ^∆∆^ *P*<0.01.

Source: elaborated by the authors.

CD3+ T lymphocytes were selected and analyzed by CD4, CD8 and CD25 staining. As shown in Fig. 6, with the increase of days of drinking DSS, the proportion of CD3+ decreased gradually (P < 0.01). Compared with the mice on the 0th day, the proportion of CD4+ gradually increased, the proportion of CD3+CD8+ cells did not change significantly, and the proportion of CD3+CD4+/CD3+CD8+ gradually increased and peaked on the 10th day. However, when drinking ultra-pure water, the proportion gradually recovered. The proportion of CD3+CD4+CD25+/CD3+CD4+ decreased gradually before the 10th day, reached the lowest value on the 10th day, and then gradually recovered.

*Effects of olsalazine on the contents of TNF-α, IL-1β, IL-2, IL-10 and IFN-γ in serum of ulcerative colitis mice*

**Figure 7:**
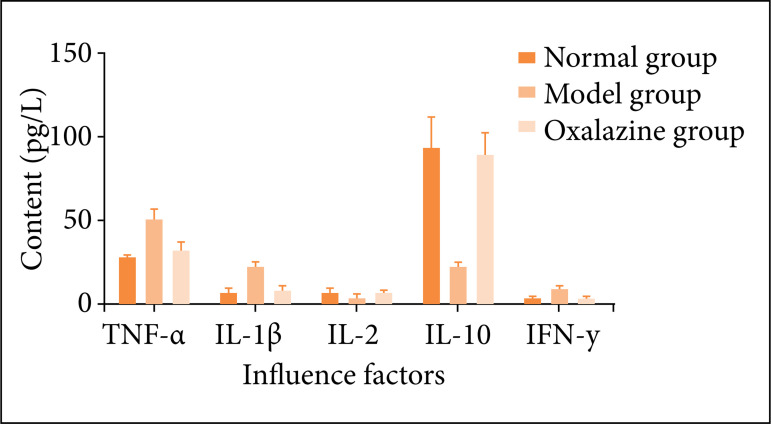
Effects of olsalazine on serum TNF-α, IL-1β, IL-2, IL-10 and IFN-γ in UC mice (Unit = ng·L^-1^; *n* = 24, *x* ± *s*). Compared with the normal group, *P < 0.05, **P < 0.01. Compared with the model group, ^∆^ *P* < 0.05, ^∆∆^ *P* < 0.01.

Source: elaborated by the authors. TNF-α: tumor necrosis factor-α; IL: interleukin; IFN-γ: interferon-γ.

Compared with the normal group, the contents of TNF-α, IL-1β and IFN-γ in the serum of the model group were significantly increased, while the contents of IL-2 and IL-10 in the serum of the model group were significantly decreased (P < 0.01 or P < 0.05). Compared with the model group, the contents of TNF-α, IL-1β and IFN-γ in serum of olsalazine group decreased significantly, while the contents of IL-2 and IL-10 increased in different degrees (P < 0.01 or P < 0.05) as shown in Fig. 7.

### Effects of olsalazine on the contents of IL-7, IL-17, IL-22, EGF and TGF in the colon of ulcerative colitis mice

**Figure 8:**
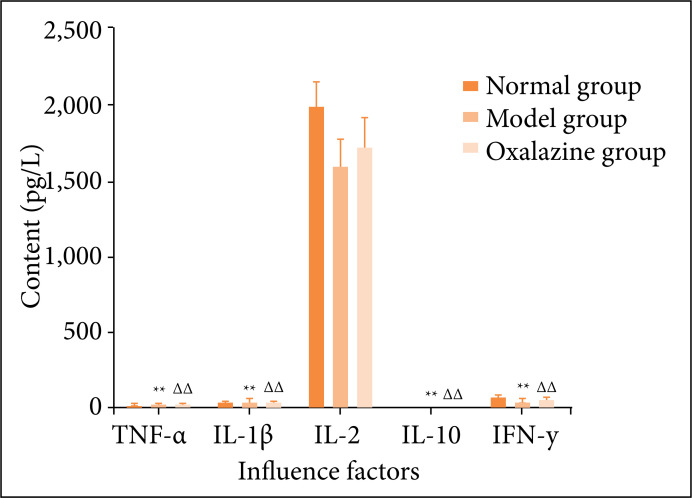
Effects of olsalazine on the contents of IL-7, IL-17 and IL-22 in colonic homogenate of ulcerative colitis mice (Unit = ng·L^-1^; *n* = 24, *x* ± *s*). Compared with the normal group, *P < 0.05, **P < 0.01. Compared with the model group, ^∆^ *P* < 0.05, ^∆∆^ *P* < 0.01.

IL: interleukin; TGF: transforming growth factor; EGF: epidermal growth factor. Source: elaborated by the authors.

Compared with the normal group, the contents of IL-17 and IL-7 in the colonic tissue of the model group increased significantly, while the contents of IL-22, TGF and EGF decreased significantly. Compared with the model group, the content of IL-7 and IL-17 in the colon of olsalazine group decreased significantly, while the contents of TGF and EGF increased significantly, as shown in Fig. 8.

## Discussion

The purpose of this study was to investigate the therapeutic effect and mechanism of olsalazine on UC in mice. It will cause a series of immune responses when the antigen invades the intestinal mucosal barrier. Intestinal epithelial cells and dendritic cells absorb the antigen and present it to immature T lymphocytes (T-lymphocyte), stimulating T cells to proliferate and differentiate into helper T cells, effector T cells and regulatory T cells. B lymphocytes are also activated, as well as secrete antibodies^13^,^14^. Activated T cells secrete a large number of cytokines, such as IL-1β, TNF-α, IFN-γ, etc., which promote the chemotaxis of neutrophils and monocytes / macrophages to gather in the inflammatory site, secrete inflammatory factors, and aggravate inflammation^15^. Some immunomodulatory cells in the body, such as Treg, Th3 and Tr1 cells, reduce the secretion of pro-inflammatory factors by IL-2 and IL-10 cells through contact inhibition in the acute stage of inflammation at the same time^16^.

The results of this experiment showed that the DAI score, CMDI score and colon HS of mice increased at first and then decreased at each time point after DSS modeling. On the 14th day, slight congestion, edema, and no obvious ulcer were observed in the colon of mice. CMDI scores and colon HS were still significantly higher than those on the first day (*P* < 0.01), indicating that the model was successfully replicated and stable. Colonic congestion, shortening, narrowing, and thickening of intestinal wall were caused by colonic mucosal barrier injury in mice after modeling. Colonic index, colonic CMDI scores and colonic HS in the model group were significantly higher than those in the normal group (*P* < 0.01). Olsalazine can inhibit the expression of TNF-α, IFN-γ, IL-1β, IL-17 and IL-7, promote the expression of IL-2, IL-22 and IL-10, restore the balance of cytokines and reduce intestinal injury in mice. At the same time, by strengthening the expression of EGF and TGF, it can accelerate the repair of intestinal injury, and further restore the animal body to normal.

## Conclusion

Sufficient and reliable data were obtained by exploring the therapeutic effect of olsalazine on DSS-induced UC in mice in this study. It was proved that olsalazine can promote the expression of IL-2, IL-22 and IL-10 by inhibiting the expression of TNF-α, IFN-γ, IL-1β, IL-17 and IL-7 in UC mice, and accelerate the repair of intestinal injury by enhancing the expression of EGF and TGF, so as to achieve the purpose of treating UC.

## Data Availability

All dataset were generated or analyzed in the current study.
